# Low-dose aspirin-associated upper gastric and duodenal ulcers in Japanese patients with no previous history of peptic ulcers

**DOI:** 10.1186/1756-0500-6-455

**Published:** 2013-11-12

**Authors:** Naohiko Kawamura, Yoshitsugu Ito, Makoto Sasaki, Akihito Iida, Mari Mizuno, Naotaka Ogasawara, Yasushi Funaki, Kunio Kasugai

**Affiliations:** 1 Department of Gastroenterology, Aichi Medical University School of Medicine, 1-1 Yazakokarimata, Nagakute, Aichi 480-1195, Japan

**Keywords:** Low-dose aspirin, Peptic ulcer, Primary prevention, Proton pump inhibitors, Risk factor

## Abstract

**Background:**

Long-term administration of low-dose aspirin (LDA) is associated with a greater risk of adverse events, including gastroduodenal ulcers. The purpose of this study was to identify the risk factors for and assess the role of medication use in the development of peptic ulcer disease in Japanese patients with no history of peptic ulcers.

**Methods:**

Consecutive outpatients receiving LDA (75 mg/day) who underwent esophagogastroduodenoscopy between January and December 2010 were enrolled. Clinical parameters, peptic ulcer history, concomitant drugs, the presence of *Helicobacter pylori* infection, reason for endoscopy, and endoscopic findings were analysed.

**Results:**

Of 226 total patients, 14 (6.2%) were endoscopically diagnosed with peptic ulcer. Age, sex, current smoking status, current alcohol consumption, endoscopic gastric mucosal atrophy, and abdominal symptoms were not significantly associated with peptic ulcers. Diabetes mellitus was more frequent (42.9% vs. 16.5%; *P* = 0.024) in patients with peptic ulcers than in those without peptic ulcers. Using multiple logistic regression analysis, co-treatment with anticoagulants or proton pump inhibitors (PPIs) was significantly associated with increased and decreased risk for peptic ulcer, respectively (odds ratio [OR], 5.88; 95% confidence interval [CI], 1.19 − 28.99; *P* = 0.03 and OR, 0.13; 95% CI, 0.02 − 0.73; *P* = 0.02, respectively). Co-treatment with additional antiplatelet agents, H_2_-receptor antagonists, angiotensin II Type 1 receptor blockers, angiotensin-converting enzyme inhibitor, 3-hydroxy-3-methylglutaryl-CoA reductase inhibitor, or nonsteroidal anti-inflammatory drugs was not associated with peptic ulcer development.

**Conclusion:**

The use of PPIs reduces the risk of developing gastric or duodenal ulcers in Japanese patients taking LDA without pre-existing gastroduodenal ulcers. However, this risk is significantly increased in both patients ingesting anticoagulants and patients with diabetes. These results may help identify patients who require intensive prophylaxis against aspirin-induced peptic ulcers.

## Background

The use of low-dose aspirin (LDA; 75 − 325 mg daily) contributes to the prevention of thrombi and emboli in patients suffering from ischemic heart disease or ischemic cerebrovascular disease [1]. However, LDA has been shown to cause gastrointestinal damage [2] and be associated with increased risk of gastroduodenal ulcers and their potentially fatal complications (e.g., gastrointestinal bleeding and perforation) [3]. In case-control studies, the odd ratios (ORs) of bleeding are in the range of 2.4 − 7.7 [4,5], which is similar to that seen in regular users of nonsteroidal anti-inflammatory drugs (NSAIDs) [6]. The mortality attributable to the use of NSAIDs or LDA is approximately 20 − 25 cases per million people, and one-third of these cases are attributable to LDA [7].

The identified risk factors for ulcer bleeding with aspirin use are history of ulcer bleeding; aspirin dose; advanced age (>70 years); concomitant use of NSAIDs or anti-coagulants; use of dual anti-platelet therapy; *Helicobacter pylori* (*H. pylori*) infection; and history of alcohol abuse, diabetes, or renal failure [6,8]. Proton pump inhibitors (PPIs) are used to decrease LDA-associated gastroduodenal mucosal and NSAID-induced injuries [9-11]. In Japan, since 2011, treatment with half-dose PPI (lansoprazole 15 mg/day) has been permitted as a medical service under health insurance for the prevention of NSAID- or LDA-induced peptic ulcers in patients in the high-risk group who have a history of peptic ulcers. Yeomans et al. [12] alleged that the use of esomeprazole 20 mg reduces the risk of developing LDA-associated gastric and/or duodenal ulcers in elderly patients without pre-existing gastroduodenal ulcers. However, there are few reports in which the use of PPIs reduced the risk of LDA-associated peptic ulcers in patients without pre-existing peptic ulcers. The aim of this retrospective study is clarify the effect of PPIs and other drugs on peptic ulcer development in Japanese patients who are taking LDA but do not have pre-existing peptic ulcers.

## Methods

This was a retrospective observation study performed at Aichi Medical University Hospital between January and December 2010. This study was approved by the ethical committee of Aichi Medical University. All endoscopic examinations were recorded digitally. Consecutive outpatients taking LDA (75 mg/day) for >3 months were enrolled, and the endoscopic findings were blindly evaluated by 1 experienced endoscopist. We defined an ulcer as a mucosal deficit > 5 mm in diameter. The exclusion criterion was a history of gastrectomy.

We recorded the following data from patient medical records: clinical characteristics, including sex, age, and smoking and drinking habits; underlying disease (hypertension, hyperlipidemia, ischemic heart disease, diabetes mellitus, cerebrovascular disease, collagen disease); peptic ulcer history; concomitant drugs (gastric agents, anticoagulants [warfarin potassium]); antiplatelet drugs (ticlopidine HCL, clopidogrel sulphate, cilostazol); NSAIDs; corticosteroids; antihypertensives; antihyperlipidemics; the presence of *H. pylori* infection; reason for endoscopy (abdominal symptoms [epigastric pain, heartburn, dysphagia, anorexia, nausea] or bleeding signs [anaemia, hematemesis, tarry stool]); and endoscopic findings.

*H. pylori* infection was determined using a rapid urease test, culture, or histology. The criterion of no pre-existing gastroduodenal ulcers was defined as no peptic ulcer history by medical record and no evidence of peptic ulcer scarring on endoscopy. Gastric mucosal atrophy was endoscopically scored on a 6-grade scale (C1, C2, C3, O1, O2, and O3; C, closed; O, open) according to Kimura and Takemoto’s classification [13]. The presence of gastric mucosal atrophy was defined as an endoscopic score of C3–O3.

## Results

A total of 226 patients (mean age, 72.0 years) were enrolled, and 14 patients (6.2%) were endoscopically diagnosed with peptic ulcers. Patient demographic and clinical characteristics are shown in Table 1. Ulcer lesions were found in the stomach of 12 patients (5.3%) and in the duodenum of 2 patients (0.9%). Age, sex, current smoking status, current alcohol consumption, endoscopic gastric mucosal atrophy, and abdominal symptoms were not significantly associated with peptic ulcers. As the underlying disease of aspirin users, diabetes mellitus was more frequent (42.9% vs. 16.5%, *P* = 0.024) in patients with peptic ulcers than in those without peptic ulcers (Table 1).

**Table 1:** Demographic and clinical characteristics of patients with and without peptic ulcer

	**Patients without ulcer**		**Patients with ulcer**			
**Variable**	**n = 212**		**n = 14**		***P *** **value**	
Mean age, years (SD)	71.9	(9.5)	72.6	(7.2)	0.785	*
≥70 years old (%)	124	(58.5)	11	(78.6)	0.168	**
Male sex (%)	128	(60.4)	12	(85.7)	0.086	**
Current smoker (%)	30	(14.2)	1	(7.1)	0.699	**
Current alcohol drinker (%)	20	(9.4)	1	(7.1)	1.000	**
Gastric mucosal atrophy (%)	98	(46.2)	6	(42.9)	1.000	**
Hypertension (%)	143	(67.5)	9	(64.3)	0.776	**
Ischemic heart disease (%)	94	(44.3)	4	(28.6)	0.281	**
Other cardiac disease (%)	20	(9.4)	1	(7.1)	1.000	**
Cerebrovascular disease (%)	30	(14.2)	0	(0.0)	0.225	**
Diabetes mellitus (%)	35	(16.5)	6	(42.9)	0.024	**
Hyperlipidemia (%)	102	(48.1)	6	(42.9)	0.787	**
Chronic renal failure (%)	14	(48.1)	2	(14.3)	0.260	**
Abdominal symptoms (%)	80	(37.7)	8	(57.1)	0.166	**

*Unpaired *t* test, **Fisher’s exact test.

On univariate analysis, the percentage of patients with peptic ulcers who were taking anticoagulants was significantly higher (28.6% vs. 9.0%; OR, 3.53; 95% CI, 1.20 − 10.36). Co-treatment with anticoagulants was significantly associated with peptic ulcers in the multiple logistic regression analysis after adjustment for age and sex (OR, 5.88; 95% CI, 1.19 − 28.99). The percentage of patients taking PPIs was significantly lower in the group with peptic ulcers than in the group without peptic ulcers (14.3% vs. 42.0%; adjusted OR, 0.13; 95% CI, 0.02 − 0.73. Co-treatment with additional antiplatelets, H_2_-receptor antagonists (H_2_RA), angiotensin II Type 1 receptor blockers, angiotensin-converting enzyme inhibitor, 3-hydroxy-3-methylglutaryl-CoA reductase inhibitor, and NSAIDs was not associated with peptic ulcers (Table 2).

**Table 2:** Association of peptic ulcer and use of other medicines among patients taking low-dose aspirin

	**Case without ulcer**		**Case with ulcer**				**Adjusted OR**	
**Medicine**	**n = 212 (%)**		**n = 14 (%)**		OR (95% CI)*		(95% CI)**	
Anticoagulant^a^	19	(9.0)	4	(28.6)	4.06	(1.16 − 14.21)	5.88	(1.19 − 28.99)
Other antiplatelet^b^	49	(23.1)	1	(7.1)	0.26	(0.03 − 2.01)	0.20	(0.02 − 1.83)
PPI^c^	89	(42.0)	2	(14.3)	0.23	(0.05 − 1.05)	0.13	(0.02 − 0.73)
H2RA^d^	36	(17.0)	1	(7.1)	0.38	(0.05 − 2.97)	0.35	(0.04 − 3.20)
Ca-blocker^e^	67	(31.6)	4	(28.6)	0.87	(0.26 − 2.86)	1.27	(0.32 − 5.14)
ARB^f^	99	(46.7)	5	(35.7)	0.63	(0.21 − 1.96)	0.45	(0.12 − 1.65)
ACE inhibitor^g^	17	(8.0)	1	(7.1)	0.88	(0.11 − 7.16)	0.39	(0.05 − 4.34)
HMG-Co A redactase inhibitor^h^	94	(44.3)	6	(42.9)	0.94	(0.32 − 2.81)	2.09	(0.53 − 8.29)
NSAID^i^	9	(4.3)	2	(14.3)	3.76	(0.73 − 19.36)	6.48	(0.75 − 56.41)

OR, odds ratio; CI, confidence interval; PPI, proton pump inhibitor; H2RA, H2-receptor antagonist; ARB, AT1-receptor blocker; ACE, angiotensin-converting enzyme; HMG-Co A, 3-hydroxy-3-methyglutaryl coenzyme A, NSAID, non-steroidal anti-inflammatory drug.

*Univariate analysis; ** Multivariate analysis adjusted for age and sex.

^a^Warfarin 1 − 5 mg/day, ^b^Ticlopidine 100 or 200 mg/day, clopidogrel 75 mg/day, cilostazol 200 mg/day, ^c^Omeprazole 10 or 20 mg/day, lansoprazole 15 or 30 mg/day, rabeprazole 10 mg/day, ^d^Famotidine 10 or 20 or 40 mg/day, ranitidine 150 mg/day, roxatidine 150 mg/day, lafutidine 10 mg/day, ^e^Nifedipine 20 or 40 mg/day, amlodipine 2.5 or 5 mg/day, ^f^Candesartan 4 or 8 mg/day, telmisartan 40 mg/day, olmesartan 20 mg/day, ^g^Imidapril 5 mg/day, ^h^Pravastatin 10 mg/day, atorvastatin 10 mg/day, ^i^Loxoprofen 60 or 120 or 180 mg/day, selecoxib 100 mg/day, naproxen 300 mg/day.

## Discussion

To our knowledge, there is only 1 prospective trial investigating the risk of primary peptic ulcer development in patients taking LDA with or without concomitant acid suppressive therapy [12]. In that trial, only patients without pre-existing peptic ulcers were enrolled, and 26-week treatment with esomeprazole at 20 mg once daily significantly reduced the risk of peptic ulcer formation in patients taking LDA for the secondary prevention of cardiovascular events. However, the patients were ≥60 years of age and tested negative for *H. pylori*. In our study, we retrospectively investigated the association between peptic ulcers and clinical parameters, including a combination of medicine, in a restricted population of patients without pre-existing peptic ulcers.

In a Japanese epidemiologic study, there was minimal biological interaction between *H. pylori* infection and NSAIDs with respect to bleeding [5], and NSAIDs and *H. pylori* infection seem to be independent risk factors for peptic ulcers and bleeding. In this retrospective study, *H. pylori* infection was observed in only 8 patients; therefore, we could not investigate the influence of *H. pylori* infection on peptic ulcer development. In the present study, previous eradication therapy was not confirmed in 216 (96.5%) patients, and 104 (46.0%) patients were found to have endoscopic gastric mucosal atrophy. This result suggests that the *H. pylori* infection rate is high. Previous studies have documented a risk of peptic ulcer complications for upper gastrointestinal bleeding [8,14-16]; however, there were only a few reports on patients without pre-existing peptic ulcers. In the present study, diabetes mellitus was more frequently observed in patients with ulcers than in those without ulcers as the causative disease of peptic ulcer in aspirin users without a history of peptic ulcers. The present results are consistent with those of former reports. Concomitant anti-coagulant therapy is significantly associated with an increased risk of peptic ulcers. However, anticoagulants have not been convincingly shown to increase the risk of ulcer development. In the present study, of the 23 patients taking concomitant anticoagulants, 9 (39%) had a bleeding rate that was higher than 40 (20%) of the 203 patients who were not taking concomitant anticoagulants. These results suggest that anticoagulants might increase the risk of LDA-induced ulcer bleeding.

In the present study, co-treatment with PPIs significantly reduced the risk of peptic ulcers. This result is consistent with that of the study by Yeomans et al. [12], a prospective, randomized, placebo-controlled trial. Moreover, in our study, there were 7 bleeding cases (6 without PPI, 1 with PPI) in 6 patients with gastric ulcers and 1 patient with a duodenal ulcer. Of the 135 patients who were not taking PPIs, 6 (4.4%) had gastroduodenal bleeding, a rate that was higher than that for placebo (1.0%) in the study by Yeomans et al. The difference may be the influence of *H. pylori* infection; however, our data are very limited and a further study is necessary to verify our findings. LDA was associated with the risk of gastroduodenal ulcers and their potentially fatal complications (e.g., gastrointestinal bleeding), even in patients without pre-existing peptic ulcers.

The identified risk factors for gastrointestinal ulcer with LDA use are as follows: a history of ulcer; aspirin dose; advanced age (>70 years); concomitant use of NSAIDs; use of dual anti-platelet therapy; *H. pylori* infection; and history of alcohol abuse, diabetes, or renal failure [6,8]. PPIs and H_2_-RA are used to decrease LDA-associated gastroduodenal mucosal lesions [9-11,17]. However, these studies included patients with a peptic ulcer history (the highest risk factor) and did not analyzed only patients without a peptic ulcer history. In patients without a peptic ulcer history, the risk factors have not yet been clarified, although they may be the same. In this study, advanced age; concomitant use of NSAIDs; use of dual anti-platelet therapy; *H. pylori* infection; and a history of alcohol abuse, or renal failure were not identified risk factors. Additionally, H_2_RA was not an identified protective factor. This outcome may have occurred as a result of our study being insufficiently powered.

Because of its retrospective design, the limitations of the present study include the absence of a diagnosis of *H. pylori* infection and ambiguous clinical information, especially with regard to medication history, including NSAIDs. In our clinic, information about anti-platelets and anti-coagulants is clear because these medications were carefully recorded habitually prior to endoscopic examinations. Because this study included only those patients without a history of peptic ulcers, a large-scale and prospective study will be necessary to clarify the risk or protective factors of LDA-associated gastroduodenal mucosal lesions.

## Conclusions

PPI therapy reduces the risk of developing gastric or duodenal ulcers in patients without pre-existing gastroduodenal ulcers. However, this risk is significantly increased in both patients who are ingesting anticoagulants and patients with diabetes. These results may help identify patients who require intensive prophylaxis against aspirin-induced ulcers. Further studies are necessary to clarify the indication of treatment with PPIs for preventing NSAID- or LDA-induced peptic ulcers in patients without a history of peptic ulcers.

## Abbreviations

LDA: Low-dose aspirin; PPIs: Proton pump inhibitors; NSAIDs: Nonsteroidal anti-inflammatory drugs; H2RA: H_2_-receptor antagonist.

## Competing interests

The authors have no competing interests to declare.

## Author’s contributions

IY, IA, MM, ON, and KK co-ordinated and collected samples from the study participants; SM was involved in editing the manuscript; and KN designed and wrote the manuscript. All authors read and approved the final manuscript.
